# Development and validation of the disease - specific problems questionnaire for patients with multiple sclerosis

**DOI:** 10.1186/s12883-021-02442-y

**Published:** 2021-10-27

**Authors:** Ali Dehghani

**Affiliations:** Department of Community Health Nursing, School of Nursing, Jahrom University of Medical Sciences, Jahrom, Iran

**Keywords:** Validation, Measurement, Global health, Questionnaire, Self-report, Multiple sclerosis

## Abstract

**Background:**

Patients with multiple sclerosis face numerous problems during their lifetime. A self-report measurement of disease - specific problems is required to be developed for patients with multiple sclerosis based on different cultural factors. Accordingly, this can advance our understanding on the disease-specific problems for care planning as well as improving coping ways and quality of life. This study aimed to develop and validate the scale of disease-specific problems of Multiple Sclerosis.

**Methods:**

This was an exploratory sequential mixed method study conducted in three phases. Correspondingly, in the first phase, the concept of disease-specific problems was defined using the content analysis approach in patients with MS. In the second phase, the item pool was generated from the findings of the first phase, and in the third phase, psychometric properties of the scale, including face, content, and construct validity and reliability, were evaluated.

**Results:**

After examining both validity and reliability, 28 items were developed in the final questionnaire. As well, by performing the factor analysis, five factors were revealed as follows: physical problems, psychological problems, emotional problems, family problems, and socio-economic problems. Internal consistency and stability of the questionnaire were calculated as 0.82 and 0.90, respectively, indicating an excellent reliability.

**Conclusion:**

The 28-item questionnaire is valid and reliable for measurement of level of disease - specific problems in Iranian people with MS.

**Supplementary Information:**

The online version contains supplementary material available at 10.1186/s12883-021-02442-y.

## Background

Multiple sclerosis (MS) is a chronic inflammatory demyelinating disease leading to the development of progressive neurological disabilities [1]. It affects people aged between 20 and 40 years old. The prevalence of MS was estimated as 1.1 million worldwide [2]. According to the MS Society of Iran, the number of people with MS is reported as 78,890 cases [3].

MS is known as one of the most important life-changing diseases because it damages the best periods of people’s life and consequently leads to disability [4]. Symptoms and problems of MS, including fatigue, pain, spasm, weakness, visual impairment, imbalance, tremor, impotence, depression, and cognitive problems, are variable and unpredictable [5]. In addition, patients with MS mostly experience several physical and psychological problems affecting their daily activities, family and social life, independence, and individual planning for the future [6, 7].

Besides the problems experienced by patients with MS worldwide, Iranian patients mostly face additional problems in having access to medications, support for rehabilitation and medical costs, etc., all of which affect the management of disease and the patients’ quality of life [8]. Some studies have previously investigated problems related to this disease in the patients; however, the majority of these studies have focused on only one aspect of these problems, including physical problems and using separate questionnaires for the same problem [9–11].

The main issue in this regard is that problems of these patients should be examined by a comprehensive, standardized, and native questionnaire. Those problems related to each dimension should also be identified using standard questionnaire, so that they can be effective on solving problems in these patients. Forbes et al. [12] in their study have developed a questionnaire to measure health-related quality of life in people with MS. In their study, the focus was only on assessing physical problems by a researcher-made questionnaire consisting of some items, including fatigue, pain, urinary-intestinal incontinence, depression, pressure ulcer, sexual problems, and employment. Indeed, no separate questions exist in this questionnaire regarding other problems in patients with MS like their family problems. Moreover, these questionnaires are used in different communities and cultures without considering any cross-cultural issue [13].

As anther similar questionnaire, measure of HRQoL was developed to assess physical, psychological, and social effects of health conditions on the individuals’ well-being. Accordingly, this questionnaire is a general tool used to differentiate between disease-specific and general conditions [14]. Although some general measures like HRQoL may capture different elements of quality of life in patients, they consist of some domains that could be in different contexts rather than being specific to a condition [15]. As well, in this field, another commonly used questionnaire is the MSQOL-54, which mainly examines physical-psychological problems and does not include the problems attributed to patients with MS in different dimensions such as family and economic problems [16]. General Health Questionnaire (GHQ-28) is also used to detect psychiatric disorders in medical practice, which has been previously validated for use among individuals with neurological disorders [17]. The Guy’s Neurological Disability Scale (GNDS) comprises 12 symptom areas related to MS such as mood, mental, bladder, and bowel [18]. The above-mentioned questionnaires mainly examine the physical and psychological problems of patients with neurological disorders. On the other hand, in the development of these questionnaires, all stages of the psychometric properties of the questionnaire have not been completed and the items of the questionnaire have also been extracted from literature review. Indeed, no specific questionnaire has been developed for the measurement of disease-specific problems in patients with MS so far.

The present study aimed to develop a valid and reliable questionnaire to assess the level of disease-specific problems in patients with MS, namely MS disease-specific Problems Questionnaire (MSPQ). Since the assessment of disease-specific problems in patients with MS can provide appropriate information regarding problem identification and making future decisions in some fields such as education, interventions, reformation, and improvement. On the other hand, the developed questionnaire must be adapted to socio-cultural context of Iran. Therefore, this study was conducted with these objectives.

## Materials and methods

### Study design and participants

This was an exploratory sequential mixed method study that was conducted in three phases and two sections of qualitative and quantitative. An exploratory sequential mixed method study includes the following steps [19]:

1. Defining the concepts
2. Formulating the items of the questionnaire
3. Developing the questionnaire
4. Testing validity and reliability of the questionnaire

**Fig. 1:**
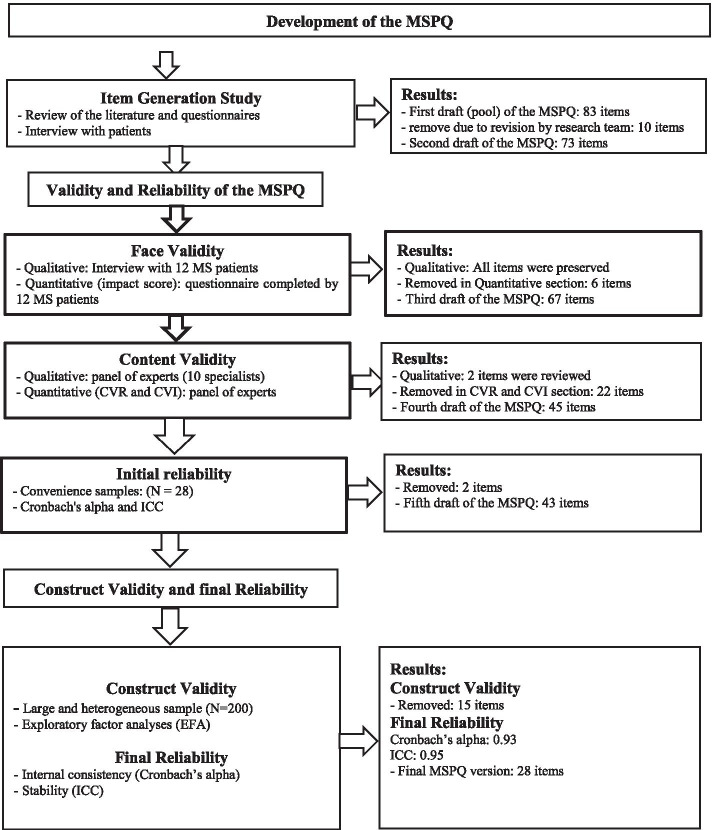
Flow diagram of the development and validation of the disease - specific problems Questionnaire in MS patients (MSPQ)

Data were collected from February 2019 to December 2020 at the MS Society in Jahrom, Iran. The study was conducted in three phases as the following (Fig. 1).

### The first phase

In this phase, the concept of disease-specific problems in patients with MS was conceptualized and then defined using the conventional content analysis. Using this method, some codes and classifications were directly extracted from the interviews with patients. Through the content analysis, the findings are interpreted by translating data in words and putting in some themes, involving some areas, including understanding, interpreting, and conceptualizing of underlying meanings of the qualitative data [20].

At this stage, 15 patients with MS were enrolled in the study. The required data were collected from the participants using semi - structured and individual interviews. The inclusion criteria were the followings: (1) definite diagnosis of MS, (2) willingness to participate in the study, (3) ability to express their experiences, and (4) at least two years past from MS diagnosis. Of note, the patients at the exacerbation stage were excluded from the study. Each interview lasted on average 45–60 min, and these interviews were conducted in the MS Society in Jahrom based on the participants’ prior agreement regarding the comfortable time. The interviews with the patients continued up to reaching data saturation. Moreover, these interviews were audio-recorded, then transcribed verbatim in MAXQDA software, Ver10 to manage coding process. Thereafter, the obtained data were analyzed using the conventional content analysis developed by the Graneheim and Lundman model [21]. Furthermore, at this stage, the initial codes were extracted, and subcategories and categories were then formed. Finally, dimensions of disease-specific problems were extracted and their concept was defined in patients with MS.

### The second phase

In this phase, the items pool was formed to develop a disease - specific problems questionnaire for patients with MS based on the following steps:

1. Dimensions extracted from the first phase of the study for disease - specific problems
2. Reviewing relevant texts in the field of disease - specific problems
3. Reviewing relevant problems questionnaires

### The third phase

In this phase, psychometric properties, including face, content, and construct validity, and reliability of the items pool, were examined in order to develop the questionnaire. These properties were as follows:

A) Face validity: The face validity was examined in two qualitative and quantitative sections. Accordingly, the qualitative section was conducted by holding individual interviews with 12 patients with MS. During these sessions, these patients were asked about the difficulty, relevancy, and ambiguity of the items, and then the questionnaire was revised based on their recommendations. For the quantitative part, these 12 patients were requested to evaluate the questionnaire and the rank each item based on its importance on a 5-point Liker scale in order to calculate ‘Item Impact Score’ (Impact Score = Frequency (%) × Importance). Finally, the impact score of 1.5 or above was considered as satisfactory [22].
B) Content validity: same as previous, content validity was evaluated in two qualitative and quantitative sections. In the qualitative part, 12 experts were asked to assess the questionnaire about its grammar, clarity of items, placement of items, and accurate scoring system [23]. In the quantitative part, both content validity ratio (CVR) and content validity index (CVI) were calculated for each one of the items of the questionnaire. The CVR of each item was then evaluated on a 3-point scale, including essential, useful, but not essential by 10 experts, based on Lawshe [24] and the modified table by Ayre and John Scally [25]. Of note, CVR varies between 1 and − 1, in a way that a higher score indicates greater agreement among panel members. Given the number of 12 experts, the items with the CVR value of 0.80 and higher were maintained. Formula of CVR is as follows: CVR = (Ne – N/2)/(N/2), where Ne is the number of panelists indicating an item as “essential” and N is the total number of panelists [26]. Using the CVI, the relevance of each item was analyzed by 10 experts on a 4-point Likert scale (scored as not relevant: 1; a little relevant: 2; somewhat relevant: 3; and extremely relevant: 4) [26]. The CVI was computed as the number of the experts giving a rating item 3 or 4 for each item divided by the total number of the experts. Values ranged from 0 to 1, and when CVI > 0.79, the item was relevant, between 0.70 and 0.79, the item needed revisions, and if the value was below 0.70, the item was eliminated [27].
C) Initial reliability: In this part, correlations between items and the questionnaire were calculated using Cronbach’s alpha and inter-item correlation coefficient (ICC).
D) Construct validity: in this part, the exploratory factor analysis (EFA) was used to determine construct validity of the MSPQ. Correspondingly, the EFA was used to determine any interrelationship between items and also to summarize the related items in a dimension [28]. In the EFA, by applying the principal axis factoring (PAF) for factors’ extraction, Kaiser- Meyer- Olkin index (KMO) was used to determine the sampling adequacy. Thereafter, Bartlett’s Test was used for the evaluation of correlation between the items of the questionnaire in order to integrate them and oblique rotation for simplifying and interpreting the factor structure through taking the eigenvalues greater than 1. The number of the participants required for performing factor analysis per each item is between 3 and 10 people [29]. The questionnaire was completed by 200 patients with MS who were selected through convenience sampling (Table 1). In this regard, Factor loadings more than 0.3 were considered as appropriate [30].

**Table 1:** Demographic characteristics of the samples in EFA section (*N* = 200)

variables		N (%)
Age (years)	Mean ± SD	36.12 ± 9.11
Duration of MS (years)	Mean ± SD	11.22 ± 4.31
Gender	male	75 (37.5)
	female	125 (62.5)
Educational status	Under diploma	42 (21)
	diploma	(30) 60
	Upper diploma	98 (49)
Number of recurring during the past year	No recurring	128 (64)
	once	40 (20)
	More than two times	32 (16)
Expanded Disability Status Scale (EDSS)	0–1.5	145 (72.5)
	2–3.5	52 (26)
	4–5.5	3 (1.5)

After factor analysis, the known-groups comparison was used for evaluating the test’s ability to discriminate between groups based on different mean scores on the test [31]. The known groups in this study were groups of MS patients with different educational levels. Thus, score of disease - specific problems was measured and compared using one-way analysis of variance one way ANOVA test in three groups of education. The convergent validity was also used for correlation between the results of two questionnaires measuring the same variable that are theoretically related [32]. The Persian version of the General Health Questionnaire (GHQ-28) [17] was employed to assess the convergent validity of the MSPQ. Thus, 200 MS patients were concurrently completed both the Persian version of the General Health Questionnaire (GHQ-28) and the MSPQ. Then, the correlation between scores of the two scales was compared using Pearson test.

E) Final reliability: Reliability of the MSPQ was determined through internal consistency and stability methods. For determinate the internal consistency, the MSPQ was completed by 20 MS patients and then Cronbach’s alpha coefficient was calculated. Alpha coefficient above 0.7 was considered as appropriate for the reliability [33]. In order to evaluate the stability of the MSPQ, a test-retest method was conducted. The MSPQ were completed by 20 MS patients at two time stages with on 2-week intervals [34]. Then, the correlation of scores between the two tests was determined with ICC. The ICC above 0.8 represents the optimal stability of the questionnaire [35].

### Statistical analysis

Statistical analyses were conducted using the SPSS version 20.0. Normality of data was confirmed through Kolmogorov-Smirnov test. Descriptive analysis, factor analysis, EFA, PAF, KMO, Bartlett’s Test, Cronbach’s alpha, ICC and Pearson test were used for data analysis.

## Results

The results of the study are presented in three phases.

### The first phase

In this phase, the concept of disease-specific problems was defined in patients with MS based on the literature review as well as the patients’ experiences using the conventional content analysis. The disease-specific problems in patients with MS are considered as a dynamic, complex, and multidimensional concept with different dimensions. Accordingly, the dimensions of disease-specific problems among MS patients include physical problems, psychological problems, emotional problems, family problems, and socio-economic problems.

### The second phase

In this phase, findings obtained from the literature review and the qualitative content analysis were employed to generate an item pool for the MSPQ scale. The item pool consists of 83 items categorized into five aspects of physical, psychological, emotional, family, and socio - economic problems. Afterward, the research team reviewed the items of the MSPQ scale to assess overlapping items, and as a result, 10 items were removed from the questionnaire and 73 items remained at last.

### The third phase

#### Face validity

In the qualitative part of the face validity, three items were modified the patients’ recommendations. Moreover, in the qualitative part of the face validity, six items were deleted due to an impact item score lower than 1.5. Therefore, 67 items finally remained in the MSPQ scale.

#### Content validity

In the qualitative part, two items were modified according to the expert panel comments. Using CVR analysis, 19 items were deleted because of obtaining CVR value lower than 0.80. In addition, three items were removed because of CVI value lower than 0.79. At last, 45 items remained in the MSPQ scale.

#### The initial reliability

The internal consistency of the MSPQ was obtained as 0.94 using Cronbach’s alpha. The correlation between items No. 13 “MS makes me to become very tired” and the whole MSPQ was 0.03, and between item No. 5 “I have spasms and muscle cramps caused by the disease”, it was obtained as −0.025. Therefore, these two items were removed due to having a correlation lower than 0.3. Eventually, 43 items remained in the MSPQ scale.

#### Construct validity

In this part, 200 MS patients included from the MS Society of Jahrom completed the 43–item MSPQ scale. The Kaiser-Meyer-Olkin (KMO) and Bartlett’s test were employed, which illustrated that the obtained data were proper for factor analysis (KMO index =0.921, χ^2^ = 5432.322, *P* < 0.001). The EFA with PAF as well as oblique rotation led to the extraction of five factors with eigenvalues greater than 1. An oblique rotation identified five latent factors, and in this regard, Table 2 shows the eigenvalues, percentage of variance for five factors, and factor loadings for the items that met the retention criteria. Therefore, 15 items were deleted from the MSPQ scale because of having factor loading lower than 0.3. Finally, 28 items and five factors remained in the MSPQ scale. Accordingly, these five factors of the MSPQ scale were as follows: factor one: “physical problems” with 9 items; factor two: “psychological problems” with 5 items; factor three: “emotional problems” with 4 items; factor four: “family problems” with 3 items; and factor five: “socio - economic problems” with 7 items. The five rotated factors explained 58.75% of the total variance. Of note, an additional file shows the developed questionnaire for this study with more details [see supplementary file].

**Table 2:** Factors, items and factor loadings of The MS disease - specific Problems Questionnaire (MSPQ −28)* (*n* = 200)

subscales	Item	Factors				
		1	2	3	4	5
**Physical problems**	I have trouble doing daily ordinary activities (bathing, wearing own clothes, etc.)	**0.802**	0.301	0.002	0.162	0.293
	I have physical pain	**0.775**	0.322	0.351	0.123-	0.220
	I have general weakness and lethargy	**0.880**	0.201	0.143	0.432	0.012-
	I have visual impairment like double vision, etc.	**0.579**	0.250	0.298	0.004	0.198
	I have an imbalance like loss of control of body movements	**0.798**	0.002-	0.005	0.321	0.213
	I have urinary and bowel incontinence	**0.467**	0.178	0.009-	0.245	0.001
	I have insomnia	**0.689**	0.234	0.432	0.006	0.015
	I have memory disorder and forgetfulness	**0.588**	0.098	0.265	0.007	0.087-
	I have tingling and numbness in the hand and leg	**0.856**	0.201	0.345	0.256	0.005
**Psychological problems**	The disease has caused negative feelings such as sadness, depression, and anxiety		**0.876**	0.201	0.444	0.093
	I feel distressed and confused	0.001	**0.895**	0.210	0.355	0.002-
	I feel feared without justified reason	0.187	**0.789**	0.421	0.220	0.045
	I feel consuming a lot of mental energy	0.002	**0.880**	0.012	0.002	0.056-
	I feel dissatisfied toward my body style	0.054-	**0.351**	0.007	0.156	0.043
**Emotional problems**	The disease makes sensitive and irritable	0.321	0.098	**0.885**	0.234	0.002
	The disease has made more introverted and indifferent toward surrounding issues	0.067	−0.045	**0.882**	0.123	0.034
	The disease has led to a decline in my emotional relationships with my spouse and family members	0.250	0.231	**0.898**	0.345	0.423
	I can’t handle my negative emotions	0.001	0.042	**0.499**	0.211	0.001-
**Family problems**	The disease has reduced my relationship with family members	0.122	0.324	0.432	**0.885**	
	The disease has reduced my role and function in the family	0.088	−0.002	0.044	**0.876**	
	The disease has caused problems in my matrimony and sexuality	0.145	0.222	0.033	**0.589**	
**Socio-economic problems**	The disease has caused problems in my job	0.542	0.057-	0.234	0.001	**0.888**
	The disease has reduced my social relationships with others	0.213	0.245	0.215	0.176	**0.880**
	The disease has reduced my ability and function in the community	0.287	0.001-	0.178	0.087	**0.689**
	I have trouble doing social activities (attending in ceremonies, etc.)	0.287	0.001-	0.321	0.098	**0.497**
	I suffer from perspective of community toward MS disease	0.213	0.321	0.002	0.045-	**0.870**
	I have trouble in providing my medication and medical treatment	0.123	0.211	0.321	0.045	**0.492**
	The lack of socio-economic supports (financial, educational, supportive, services, etc.) led to problems for me.	0.159	0.054-	0.301	0.034	**0.850**
**Eigenvalue**		**9.250**	**7.450**	**5.43**	**3.38**	**1.894**
**Percentage of variance**		**21.219**	**17.456**	**12.232**	**5.132**	**2.711**

Thereafter, the items of the MSPQ were rated on a five-point Likert-type scale from 1 to 5, 1 = never, 2 = rarely, 3 = sometimes, 4 = often, and 5 = always.

In order to assessment discriminating of the MSPQ in known-groups comparison was used from educational levels. Based on some researches [36, 37], MS patients with lower educational level have more problems. In the present study, patient’s educational level was classified in three levels including under diploma, diploma and upper diploma. The results of one way ANOVA showed a significant difference between groups (*P* value = 0.001). The results of Turkey’s post-hoc test showed disease - specific problems higher is in patients with under diploma educational level than other two groups. The result of correlation between the MSPQ and the Persian version of the General Health Questionnaire (GHQ-28) with Pearson test was 0.561 (*P* value <0.001), which represents optimal convergent validity.

#### Final reliability

Cronbach’s alpha for the 28-item MSPQ scale was 0.82, which showed optimal internal consistency. The ICC between test and retest measurements was 0.90, which indicated an acceptable stability of the MSPQ scale over the time. Cronbach’s alpha and ICC were determined for five factors that are shown in Table 3. For stability through test–retest analysis, Spearman’s correlation coefficient was reported 0.87.

**Table 3:** Descriptive statistics and reliability measurements of the MSPQ -28

Factors	Subscales	Number of items	Cronbach’s Alpha	ICC (95% CI)	Spearman’s Correlation Coefficient (*n* = 20)	*p*-Value
1	Physical problems	9	α = 0.74	ICC = 0.81	0.89	0.001
2	Psychological problems	5	α = 0.77	ICC = 0.87	0.91	0.001
3	Emotional problems	4	α = 0.80	ICC = 0.91	0.85	0.001
4	Family problems	3	α = 0.79	ICC = 0.88	0.82	0.001
5	Socio-economic problems	7	α = 0.74	ICC = 0.85	0.92	0.001
	MSPQ	28	α = 0.82	ICC = 0.90	0.87	0.001

*CI* confidence interval

## Discussion

The present study attempted to develop and validate a scale for disease-specific problems in patients with MS. Furthermore, since the objective measure ofthe disease-specific problems do not appear to capture the patient’s experiences completely, self-reports were employed in this study. The result show that the MSPQ scale is a reliable and valid instrument for the evaluation of the disease-specific problems. Our approach to create a new instrument was multifaceted and iterative using both qualitative and quantitative methods in its development. Since no instrument has been designed in Iran so far to evaluate the disease-specific problems and to evaluate the feedback to health personnel in a semi-structured model of problems identification and care planning, it was crucial to develop and validate an instrument used to evaluate the important dimensions of the disease-specific problems and to provide an opportunity for giving feedback to health personnel.

Different studies have previously used various questionnaires to assess the problems of patients with MS such as the measurement of fatigue using FSS scale [38]; stress, anxiety, and depression using DSAA 21 scale [39]; and physical problems using several questionnaires [12, 40, 41]. It was indicated that the MSPQ scale could be used to evaluate all the disease-specific problems, including physical, psychological, emotional, family, and socio – economic problems in patients with MS in a single questionnaire. In fact, it was attempted to eliminate weaknesses of other instruments in this instrument. The advantages of an overall MSPQ scale are as follows: it gives a holistic picture, as well as information on the impact of MS disease on patients. As an example, Forbes et al. [12] have introduced an instrument regarding health problems, which has been repeatedly used in various studies, but it does not evaluate some areas of the disease-specific problems such as emotional, family, and socio – economic problems, so it cannot be used for those MS patients with multidimensional problems. Besides physical problems, many people with MS experience some other problems, including the history of disorders in family and social relationships, dysfunction, social role, and occupational problems, which require attention and consideration [6]. Patients with MS mostly experience several difficulties in emotion regulation, psychological condition, and providing medical costs that predict poorer quality of life. These findings indicate that emotional and psychological outcomes controlling skills should be investigated with more details when considering interventions to enhance well-being among MS patients [42]. As well, patients with MS experience more difficulties in emotion and psychological conditions’ regulation compared to healthy people. Mediation analyses indicated that depression could mediate the emotion regulation difficulties during MS course. Therefore, difficulties in emotion regulation could help in predicting poorer psychological and social quality of life in MS patients [43].

In the present study, a positive significant correlation was found among the number of recurrences during the past year, Expanded Disability Status Scale (EDSS), and the dimension of the MSPQ. Based on results of the study by Nortvedt et al., the correlation between the sexual summary scale and EDSS was calculated as 0.24 [10]. Correspondingly, these findings are consistent with the results of studies by Pike et al. [1] and Dehghani et al. [44]. In addition, a negative significant correlation was observed among the duration of MS (years), educational level, and the dimension of the MSPQ. In consistent with these results, the findings of Simeoni et al.’s study showed that the MusiQoL dimension scores were significantly higher for patients with a greater educational level compared to those having a moderate or low educational level [36].

One of the strengths of this study was that the MSPQ was developed using both inductive and deductive methods. Its psychometrics properties have also been examined through face, content, and construct validity, as well as reliability (internal consistency and stability). The MSPQ is a 28-item short questionnaire that can be responded by patients with MS in a short time (10–15 min). The greatest strengths of this study were the design and development of a context-based health condition, in order to assess Iranian MS patients’ disease-specific problems.

The lengthy and ongoing processes in the development, validation, and evolution of a new questionnaire as well as suffering scale of self-report were the current study’s limitations. Another limitation was that it was not possible to gather objective data on the patients’ disability levels, which may be known as a mediating factor in the disease-specific problems of patients. Another important limitation of the study was the lack of measures derived from already validated questionnaires to investigate the concurrent/divergent validity of the new developed questionnaire.

## Conclusion

In this study, the five-dimension MSPQ was developed as a short self-report scale for measurement of disease - specific problems in Iranian people with MS. The MSPQ is a valid, reliable and context-based scale, which can be used in education, research, care management, needs assessment, and support services.

## Supplementary Information

**Additional file 1.** The disease - specific problems Questionnaire in Multiple Sclerosis Patients. In this section, a number of 28 items developed questionnaire to assess disease - specific problems in MS patients are listed in a table.

## Data Availability

All data generated or analyzed during this study are included in this published article. Further data set could be obtained on request if required through corresponding author with email: ali.dehghani2000@gmail.com.

## Abbreviations

MS: Multiple sclerosis
HRQoL: Health-related quality of life
MSQOL-54: Multiple sclerosis quality of life
MSPQ: Multiple Sclerosis disease - specific Problems Questionnaire
one way ANOVA: One-way analysis of variance
ICC: Intraclass correlation coefficient
PAF: Principal axis factoring
KMO: Kaiser- meyer- olkin
CVR: Content validity ratio
CVI: Content validity index
EFA: Exploratory factor analyses
MusiQoL: Multiple Sclerosis (MS) International Quality of Life
