# Supplementary material for: Development and validation of the disease - specific problems questionnaire for patients with multiple sclerosis
Source: BMC Neurol. 2021 Oct 27;21:415. doi: 10.1186/s12883-021-02442-y (PMC8555153; doi:10.1186/s12883-021-02442-y)
Supplement: Supplementary file 1 — Additional file 1. The disease - specific problems Questionnaire in Multiple Sclerosis Patients. In this section, a number of 28 items developed questionnaire to assess disease - specific problems in MS patients are listed in a table. [file 12883_2021_2442_MOESM1_ESM.docx]

Supplementary Material

**Supplementary information**

**File name:** Additional file 1

**Title of data:** The disease - specific problems Questionnaire in Multiple Sclerosis Patients

**Description of data:** In this section, a number of 28 items developed questionnaire to assess disease - specific problems in MS patients are listed in a table.

**Scoring and interpretation:**

The items of the MSPQ were rated on a five-point Likert-type scale, 1 = never, 2 = rarely, 3 = sometimes, 4 = often and 5 = always. For each individual, the score of each dimension was obtained by computing the sum of the item scores of the dimension. All dimension scores were linearly transformed to a 28–140 scale. The total score of the MSPQ was computed as the sum of the dimension scores. Higher scores indicate more disease - specific problems.

**Time of administration:** 10–15 min

| **subscales** | **Item** | never | rarely | sometimes | often | always |
| --- | --- | --- | --- | --- | --- | --- |
| **Physical problems** | I have trouble doing daily ordinary activities (bathing, wearing own clothes, etc.) |  |  |  |  |  |
|  | I have physical pain |  |  |  |  |  |
|  | I have general weakness and lethargy |  |  |  |  |  |
|  | I have visual impairment like double vision, etc. |  |  |  |  |  |
|  | I have an imbalance like loss of control of body movements |  |  |  |  |  |
|  | I have urinary and bowel incontinence |  |  |  |  |  |
|  | I have insomnia |  |  |  |  |  |
|  | I have memory disorder and forgetfulness |  |  |  |  |  |
|  | I have tingling and numbness in the hand and leg |  |  |  |  |  |
| **Psychological problems** | The disease has caused negative feelings such as sadness, depression, and anxiety |  |  |  |  |  |
|  | I feel distressed and confused |  |  |  |  |  |
|  | I feel feared without justified reason |  |  |  |  |  |
|  | I feel consuming a lot of mental energy |  |  |  |  |  |
|  | I feel dissatisfied toward my body style |  |  |  |  |  |
| **Emotional problems** | The disease makes sensitive and irritable |  |  |  |  |  |
|  | The disease has made more introverted and indifferent toward surrounding issues |  |  |  |  |  |
|  | The disease has led to a decline in my emotional relationships with my spouse and family members |  |  |  |  |  |
|  | I can't handle my negative emotions |  |  |  |  |  |
| **Family problems** | The disease has reduced my relationship with family members |  |  |  |  |  |
|  | The disease has reduced my role and function in the family |  |  |  |  |  |
|  | The disease has caused problems in my matrimony and sexuality |  |  |  |  |  |
| **Socio-economic problems** | The disease has caused problems in my job |  |  |  |  |  |
|  | The disease has reduced my social relationships with others |  |  |  |  |  |
|  | The disease has reduced my ability and function in the community |  |  |  |  |  |
|  | I have trouble doing social activities (attending in ceremonies, etc.) |  |  |  |  |  |
|  | I suffer from perspective of community toward MS disease |  |  |  |  |  |
|  | I have trouble in providing my medication and medical treatment |  |  |  |  |  |
|  | The lack of socio-economic supports (financial, educational, supportive, services, etc.) led to problems for me. |  |  |  |  |  |
